# Rationale and study protocol to evaluate the SunSmart policy intervention: a cluster randomised controlled trial of a primary school-based health promotion program

**DOI:** 10.1186/s12889-015-1371-8

**Published:** 2015-01-31

**Authors:** Dean A Dudley, Matthew J Winslade, Bradley J Wright, Wayne G Cotton, Jackie L McIver, Kirsten S Jackson

**Affiliations:** Faculty of Human Sciences/School of Education, Macquarie University, North Ryde, 2109 Australia; School of Teacher Education, Charles Sturt University, Panorama Ave, Bathurst, 2795 Australia; Faculty of Education and Social Work, The University of Sydney, Sydney, 2006 Australia; Skin Cancer Prevention Unit, Cancer Council New South Wales, Dowling St, Woolloomooloo, 2011 Australia

**Keywords:** Health promoting schools, Skin cancer, Paediatric, Preventive medicine

## Abstract

**Background:**

Previous evaluations of the SunSmart Program have supported the link between a written sun protection policy and improved sun protection behaviours in New South Wales (NSW) primary schools. However these evaluations have relied on self-reported data and research suggests that direct observations are required to better represent schools’ usual sun protective practices.

**Methods/Design:**

Data will be collected in the summer months of 2014, 2015, and 2016 as part of an 18-month cluster-controlled trial in NSW primary schools (n = 20). Researchers will conduct three direct observations to record students’ hat use and teachers’ use of sun protective measures during recess and lunch periods in each school. Researchers will also record the volume of sunscreen that the Year 6 classes in each school utilise over the term. At the conclusion of baseline data collection, five schools will be randomised into an intervention group that will work with researchers to develop a policy-driven intervention to improve sun safety behaviour in NSW primary schools.

**Discussion:**

An initial review of relevant Australian and New Zealand literature suggests that provision of policy support is likely to improve school sun protection practices; however there is no suggested model for this support. This will be the first objective analysis of sun safe behaviours leading to a policy-driven intervention conducted in Australian primary schools since the 1990s, and will inform the future direction of sun safety in our schools.

**Trial registration:**

Australian and New Zealand Clinical Trials Register ACTRN12614000926639 Registered 28^th^ August 2014.

## Background

Australia has among the highest incidence of skin cancer in the world with approximately 750,000 treatments for melanoma and skin cancer every year [1]. Protecting skin from overexposure to ultraviolet (UV) radiation is the simplest and most effective way to reduce the risk of developing melanoma and other skin cancers [2,3]. The World Health Organization (WHO) [4] suggests that school sun protection programs are the key to skin cancer prevention. The New South Wales Department of Education and Communities (NSWDEC) has demonstrated its commitment to sun safety, releasing updated Sun Safety for Students guidelines [5], which strongly encourage each school community to implement a comprehensive Sun Safety Action Plan.

Cancer Council New South Wales (CCNSW) is a not for profit research and advocacy organisation for all forms of cancer within New South Wales (NSW). The SunSmart Program is their flagship skin cancer prevention program, and supports primary schools to develop and implement comprehensive sun protection policies. The program is based on the World Health Organization’s Sun Protection and Health Promoting Schools principles [6] It applies these principles by; a) negotiating a school-endorsed sun protection policy with schools and the systems that govern them, b) providing sun protection education resources, c) advocating for a healthy and sun-safe school environment that include the scheduling of outdoor activities, providing shaded play and recreation areas, encouraging sun protective clothing, providing and encouraging the wearing of sunscreen and encouraging the role modelling of sun-safe behaviours by teachers and other significant adults at schools, and d) encouraging community and family involvement in sun protection behaviour and awareness.

Previous national and state surveys of school sun protection policies and practices demonstrate that being a member of the SunSmart Program improves sun protection practices in primary schools [7,8]. The national survey of Sun Protection Policy and Practice in Primary Schools demonstrated an association between a written sun protection policy and more effective sun protection practices [8]. However survey results also demonstrate there are opportunities to embed sun protection practices more broadly in primary schools; in particular students’ use of sun-safe hats and sunscreen, and teacher role modelling of positive sun protection practices [8].

A review of relevant Australian and New Zealand literature suggests that provision of policy support is likely to improve school sun protection practices [9,10]. Change agents (project champions like teachers) are suggested as one potential approach for improved policy implementation [11,12]. Furthermore, any policy intervention must consider the nuance and idiosyncrasies of the school and the communities they serve. Given the complexity of the school setting, intervention strategies will need to be innovative and consider issues of design and measurement in dealing with school-based samples.

In 2014 more than three-quarters of NSW primary schools are signatories to the SunSmart Program [13]. Previous evaluations of school sun protection policies have stated the data supporting the link between sun-protection policies and observations of sun protective behaviour at primary schools are lacking [14]. This evaluation by Turner and colleagues also called for research involving independent assessment of policies and direct unannounced observations of behaviour to better represent usual sun protective practices (rather than self-reported data). This study seeks to build on the body of knowledge regarding sun protection behaviours in primary schools. The study will use a novel application of an existing objective observational tool to collect data on key sun-safe practices in schools that are members of the SunSmart Program, and develop and evaluate practical strategies to support schools in improving their sun protection policy.

## Methods/Design

### Study design

The SunSmart Evaluation and Policy Intervention study is an 18-month primary school-based intervention and will be evaluated using a cluster randomised controlled trial. Ethics approval has been sought and obtained from an Australian University Human Ethics Committee (HREC 2014/062) and the New South Wales Department of Education and Communities (SERAP: 2014148). The SunSmart Evaluation and Policy Intervention study is registered with the Australian and New Zealand Clinical Trials Registry (ACTRN12614000926639). The study protocol was also reviewed internally by the research committees of the funding agencies.

Following the initial recruitment processes, researchers will conduct baseline assessments at participating schools. The design, conduct and reporting of this study will adhere to the Consolidation Standards of Reporting Trials (CONSORT) guidelines for a cluster randomised controlled trial [15]. Principals, teachers and parents will provide written informed consent.

### Sample size calculation

Power calculations were conducted to determine the sample size and number of observations required to detect changes in the outcome of students wearing sun protective headwear in a cluster design. Calculations assumed baseline-posttest expected effect size gains of *d* = 0.4/r = 0.19 and were based on 80% power, with alpha levels set at p < 0.05. Using the standard deviation (SD) of change of SD = .5, it was calculated that the study required 50 observations within the intervention and control groups would provide adequate power to detect a between group difference of *d* = 0.4 across the school day.

### Recruitment and study participants

To be eligible to participate in the study, schools must be government primary schools and be a current signatory to the SunSmart Program in the Greater Western Sydney Region, NSW, Australia (Approx 33.75 deg S, 150.70 deg E).

All eligible schools (n = 167) will be sent an initial email with an invitation to participate in the study. CCNSW and NSWDEC will also identify a short-list of schools that may be receptive to participating in the study (n = 40). Schools that respond to the initial email and the short-listed schools will be pooled and receive a follow up call in random order from the project researchers to ascertain whether they would like to participate in the study. The first twenty schools that demonstrate interest will then be recruited into the study.

**Figure 1:**
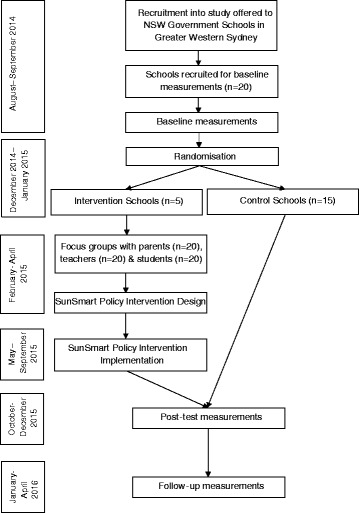
**Flowchart of study.**

Randomisation into intervention and control group will occur after baseline assessments. A simple computer algorithm will be used to randomly allocate schools to either control (n = 15) or the treatment (n = 5) conditions by an independent researcher not involved in the study. This method will ensure all schools have the same likelihood of allocation into one of the two study arms. Trained research assistants and project researchers will conduct all assessments and perform focus groups and interviews. All researchers will complete training sessions prior to assessment to maintain consistency and where possible, the same assessors will be used at baseline, post-test and follow-up. Figure 1 shows the flow of participants through the study.

### Intervention design

After randomisation, researchers will conduct focus group discussions with parents, Stage 3 (year 5 and 6) students, and individual interviews with teachers. This qualitative research will aim to investigate the results of the baseline data collection and inform the intervention design with these schools. These focus groups and interviews will unpack the positive and negative aspects of the school’s utilisation of sun-safe hats, sunscreen and staff role modelling of sun-safe behaviours.

Interviews and focus groups will also investigate schools’ understanding of being ‘SunSmart’ and other aspects of the existing school sun protection policy; including sun-safe school uniforms, the provision of shade, scheduling of outdoor activities, sun protection within the school curriculum and finally, inclusion of the local community into school sun-safe practices. This broader context will allow analysis of the relative importance of the three specific study outcomes and other factors that may have influenced the baseline results.

Data from this formative research will be analysed using coding and intra and inter-textual analysis to ascertain common themes across schools [16]. Formative interview data will be utilised to refine the design and enactment of the intervention.

The intervention development and implementation will be based on Social Cognitive Theory (SCT) [17] which focuses on the interplay between personal, behavioural, and environmental factors. The personal factors identified in SCT (such as the thoughts, emotions and biological dispositions of an individual) will be addressed by the intervention program by influencing the value systems that may reinforce the low emphasis on the school sun protection policy. The nuanced behavioural factors associated with sun protection behaviours, such as the wearing of hats and sunscreen and playing in shaded areas also need to be identified objectively and addressed within the intervention program. Finally, the environmental factors (both social and physical) that often exist beyond the immediate control of individual schools need to be identified and addressed in order to remove potential barriers and to provide opportunities for social support. An example of influencing this factor may include the utilising and modifying of existing school infrastructures that were not used by the school in order to make them more accommodating for the sun-safe practices without any additional financial outlay.

The intervention will also be based on the key principles of the WHO Health Promoting Schools (HPS) Framework [18] that focuses on the connection between formal curriculum, school ethos and the school-home-community partnership. By addressing these overlapping and interconnecting components, schools are more likely to provide comprehensive and effective health promotion throughout the entire school community. The formal curriculum of the HPS framework refers to the teaching and learning programs provided by the school, and advocates these be distributed in a variety of subjects and classes rather than just traditional health education classes [19]. A school ethos is derived by the components put in place which construct its values and atmosphere, including organisation, environment, policies and procedures [19]. The partnership between the school and local community is a vital component for school health promotion. By incorporating families and the community into the life of the school, a supportive learning and healthy environment can be established [19].

In collaboration with CCNSW and each of the primary schools assigned to the intervention group, a policy driven intervention will be rolled out over the course of two school terms (June-December 2015). Post-test data will be collected during the latter half (October-December 2015) of the intervention implementation with follow-up data collected after the schools return from summer break between January and April 2016.

### Outcomes

Evaluation of the SunSmart Policy Intervention will involve a variety of instruments to report on sun protection behaviours that occur in primary schools. Trained research assistants who will be blind to the control or intervention allocation of the schools will conduct all assessments. All sun protection behaviour practices will be measured at baseline, post-test (12 months), and follow-up (15 months).

### Wearing of headwear and prevailing environmental conditions

The primary outcome will be children’s wearing of a sun protective headwear during break periods of the school day (i.e. recess and lunch). Currently no direct observation instruments to record sun-safe behaviours of children in school settings exist. However several direct observation tools of physical activity do exist, and one of these will be adapted to capture sun-safe behaviour data in children. The System for Observing Play and Recreation in Communities (SOPARC) is based on momentary time sampling techniques in which systematic and periodic scans of individuals and contextual factors within predetermined target areas are made [20]. iPad tablets (Apple Inc, USA) installed with the iSOPARC Application Version 1.75 (CIAFEL, Portugal: http://ciafel.fade.up.pt) will be used to provide an objective measure of the wearing of hats by children during recess and lunch.

During a scan each subject is electronically coded and identified by; sex (male or female), intensity of activity (Sedentary, Walking, or Very Active), and whether they are a Child, Teen, Adult or Senior. For this study, given all the subjects will be children, the third battery of coding (Child, Teen, Adult or Senior) will be changed to detect whether the student is Unprotected (no hat), Partially-Protected (wearing a baseball cap), Fully-Protected (wearing a 360 degree brim; broad-brimmed or bucket hat) or Fully-Protected (wearing a peak cap with back flap; legionnaire hat).

Separate scans are made for females and males, and simultaneous entries are also made for time of day, temperature, UV radiation level, area accessibility, area usability and presence of supervision. Each observation is conducted twice during the recess and lunch breaks for both females and males. This results in each school having 24 observations conducted at each data collection period (i.e. baseline, post-test and follow-up).

Direct observations will be made in designated Target Areas that represent all standard locations likely to provide opportunities for students to have sun exposure during their recess and lunch periods (e.g. play equipment or outdoor sporting fields/courts). These Target Areas (one shaded and one non-shaded) will be predetermined and identified for observations prior to baseline assessments. A map will be provided to identify Target Areas and a standard observation order established for each school. Additional Target Areas may be added by observers on site and then documented.

During occasions of high student density, Target Areas will be subdivided into smaller Scan Spaces so that accurate measures can be obtained. Observers will use standard court or field markings to determine appropriate Scan Spaces within each Target Area. Data from these smaller spaces will be summed to provide an overall measure for each Target Area. A decision to subdivide a Target Area depends upon the (1) number of students in the area and (2) the type of student activity.

Additional data recorded prior to the direct observation scans includes;

1. Temperature and UV level at the start and end of the observation period; 2. Whether the observation was made at recess or lunch; 3. Start and finish times of recess and lunch; and 4. The condition of the target area. This is coded as follows:

A = Area is accessible (e.g. not locked or rented to others).

U = Area is usable for activity (e.g. is not excessively wet or windy).

SS = Area is supervised by designated school personnel who are role modelling sun safe behaviour (e.g. wearing a broad brimmed hat, wearing a shirt with a collar and minimum short sleeves, sunglasses, using shade).

SN = Area is supervised by designated school personnel who are not role modelling sun safe behaviour.

O = Organised physical activity (i.e. scheduled, with leadership by school personnel apparent) is occurring in the area (e.g. intramurals, interscholastic practices, fitness stations).

E = Equipment provided by the school or other agency is present (e.g. balls, jump ropes). This will not be coded as present if the only equipment is permanent (e.g. basketball hoops).

C = Covered area (e.g. the activity is taking place in an area where at least 50% of the target area is covered with shade provided by a permanent or natural feature such as shade cloth or tree foliage).

Researchers will record whether the teacher supervising activities in the Target Area is role-modelling sun protective behaviour. An observation note will be added to the final iSOPARC data on whether the teacher is wearing a) a hat (broad brimmed or baseball cap), b) sunglasses or other appropriate eye protection (i.e. transition lensed optical glasses, and c) a sleeved shirt and collar.

### Wearing of sunscreen

The secondary outcome will be children’s wearing of sunscreen during the school day. Currently no direct observation instruments to record the wearing of sunscreen exist. As a proxy measure of children wearing sunscreen, Grade 6 classes in control and intervention schools will be provided with one-litre containers of sunscreen for each class free of charge. Sunscreen will delivered to, and be collected from schools on the same day in order to ensure the period of use is the same in all schools; 50 days (10 school weeks) per interval. Each of the containers will their have branding removed but maintain their active ingredient information as required by the Therapeutic Goods Administration (TGA) [21]. A laminated instruction card will be attached to each bottle requesting classroom teachers to use the sunscreen as they normally would with their students.

The consumption of sunscreen from these containers will be recorded at the conclusion of the baseline, post-test and follow-up phases and will be replaced with full containers when they reach approximately 100 mL of sunscreen or less remaining.

In order to determine sunscreen consumption, 20 similar pump packs of sunscreen will be weighed to record an average starting weight for comparison. Consumption will then be analysed at the end of each data collection phase by comparing the net weight loss each pump pack of sunscreen issued to each school and the student number enrolled in Grade 6 classes who were issued the sunscreen.

### Statistical methods

Statistical analysis of the primary and secondary outcomes will be conducted with linear mixed models using SPSS statistics version 20 (IBM SPSS Statistics, 2012) and alpha levels will be set at *p* > 0.05.

The models will be used to assess the impact of treatment (SunSmart Policy Intervention or Control), time (baseline, post-test and follow-up) and the group-by-time interaction, these three terms forming the base model. The models will be specified to adjust for the clustered nature of the data and will include all randomised participants in the analysis. Mixed models are robust to the biases of missing data and provide appropriate balance of Type 1 and Type 2 errors [22]. Mixed model analyses are consistent with the intention-to-treat principle, assuming the data are missing at random [23]. Sex, temperature/UV, time of day, type of period (recess or lunch) and conditions of target area (as previously outlined) will be included as covariates in the models.

### Qualitative methods

The focus groups and interviews of Stage 3 students, parents and teachers will be used for the intervention design and will be digitally recorded with the participants’ consent and transcribed verbatim into a Microsoft Word document. Two computer programs (NVIVO 10 and Leximancer) will be used to assist with the organisational aspects of data analysis. Analysis will be conducted by members of the research team using a standard general inductive approach to qualitative analysis with NVIVO 10 and a specialised method of transforming lexical co-occurrence information from natural language into semantic patterns for qualitative analysis with Leximancer. The algorithms used by Leximancer are statistical, but they employ nonlinear dynamics and machine learning methods and have been validated, deemed stable, and reproducible [24].

## Discussion

The primary aim of this study is to evaluate the impact of a SunSmart policy intervention on children’s daily school sun protection behaviours. The study will use a novel evaluation strategy that directly observes the behaviour of children and teachers in their natural school environments. Researchers will also monitor sunscreen consumption over a ten-week time period in Grade 6 students during three summer intervals.

Previous school-based health promotion intervention studies have highlighted the importance of teacher and parental behaviour on health related intervention outcomes in primary school children [25]. A critical aspect of this study is that teachers, parents and the students themselves will be involved in the intervention design phase. Teacher, student and community ownership of intervention design has been identified in previous studies as a desirable factor to improve the effects of school-based interventions [26]. This ownership of the program has the potential to lead to greater sustainability of increased sun protection practices and enable teachers to integrate effective sun protection policies into the school day.

A clear strength of this study is the rigorous evaluation process including quantitative and qualitative measures to explore program feasibility and potential efficacy. The empirically robust design with baseline, post-test and follow-up measures will help examine the views of participants (teachers, parents and students), and help distinguish between an intervention that is poorly designed and one that may be poorly delivered [27]. We consider this a necessity in this study due to the multi-site delivery.

To our knowledge, no previous interventions have reported the effects of a policy-based intervention on sun protection behaviour outcomes using direct observation measures. Significantly, our study will be capable of collecting a host of covariates on the sun safety behaviour of primary school children namely daily temperature, period of UV exposure during recess and lunch periods, size of play area, nature of play children undertake and teacher role-modelling behaviour.

An additional study strength is the use of an objective measure of behaviour. Direct observation has a number of advantages over other possible measurement tools [28]. First, it is an objective method that provides contextually rich data to identify other factors related to sun protection behaviours (e.g., physical and social factors). Second, it can provide information on the type of hats being worn by children. The main disadvantage of direct observation is the time-intensive nature of observer training and data coding. The use of a direct observation instrument like iSOPARC is advantageous when working with children because unlike self-report measures of behaviour, they help eliminate language and literacy difficulties, recall bias and social desirability bias [29]. As research assistants will be blind to the randomisation of schools there is little to no chance of any bias impacting on the observations.

The findings of study will provide valuable information for other research groups looking for evidence based research on sun protection behaviours of primary school students in NSW. School-based sun behaviour interventions are infrequent, and seldom published in peer-reviewed journals. This study has the potential to change school policy and practice in relation to sun safety integration and enhance a range of key health promoting behaviours in schools.

## Abbreviations

NSW: New South Wales
UV: Ultraviolet
WHO: World Health Organization
NSWDEC: New South Wales Department of Education and Communities
CCNSW: Cancer Council NSW
SERAP: State Education Research Approvals Process
ACTRN: Australian and New Zealand Clinical Trials Registry Number
CONSORT: Consolidation Standards of Reporting Trials
SCT: Social Cognitive Theory
HPS: Health Promoting Schools
SOPARC: System for Observing Play and Recreation in Communities
TGA: Therapeutic Goods Administration
SPSS: Statistical Package for the Social Sciences
